# Computed Tomography of the Head Before Lumbar Puncture in Adults With Suspected Meningitis in High–HIV Prevalence Settings

**DOI:** 10.1093/ofid/ofae565

**Published:** 2024-09-26

**Authors:** James Milburn, Christopher G Williams, Kwana Lechiile, Keatlaretse Siamisang, Leah Owen, Ezekiel Gwakuba, Thandi Milton, Tichaona Machiya, Tshepo Leeme, Hannah E Barton, Ponego Ponatshego, Kaelo K Seatla, Gerald Boitshepo, Rachita Suresh, Ikanyeng Rulaganyang, William Hurt, Samuel Ensor, Kebatshabile Ngoni, Ronan Doyle, Daniel Grint, Wallace T Miller, Mark W Tenforde, Madisa Mine, David M Goldfarb, Margaret Mokomane, Joseph N Jarvis

**Affiliations:** Botswana Harvard Health Partnership, Gaborone, Botswana; Department of Clinical Research, Faculty of Infectious and Tropical Diseases, London School of Hygiene and Tropical Medicine, London, UK; Botswana Harvard Health Partnership, Gaborone, Botswana; Botswana Harvard Health Partnership, Gaborone, Botswana; Botswana Harvard Health Partnership, Gaborone, Botswana; Botswana-UPenn Partnership, Gaborone, Botswana; School of Allied Health Professions, University of Botswana, Gaborone, Botswana; Botswana-UPenn Partnership, Gaborone, Botswana; Microbiology Department, Princess Marina Hospital, Gaborone, Botswana; Botswana Harvard Health Partnership, Gaborone, Botswana; Botswana-UPenn Partnership, Gaborone, Botswana; Botswana Harvard Health Partnership, Gaborone, Botswana; Botswana Harvard Health Partnership, Gaborone, Botswana; Department of Radiology, University of Botswana, Gaborone, Botswana; Botswana Harvard Health Partnership, Gaborone, Botswana; Botswana-UPenn Partnership, Gaborone, Botswana; Botswana-UPenn Partnership, Gaborone, Botswana; Botswana Harvard Health Partnership, Gaborone, Botswana; Botswana Harvard Health Partnership, Gaborone, Botswana; Department of Clinical Research, Faculty of Infectious and Tropical Diseases, London School of Hygiene and Tropical Medicine, London, UK; Department of Infectious Disease Epidemiology and International Health, Faculty of Epidemiology and Population Health, London School of Hygiene and Tropical Medicine, London, UK; Department of Radiology, University of Botswana, Gaborone, Botswana; Botswana-UPenn Partnership, Gaborone, Botswana; National Health Laboratory, Gaborone, Botswana; Department of Pathology and Laboratory Medicine, University of British Columbia, Vancouver, Canada; School of Allied Health Professions, University of Botswana, Gaborone, Botswana; Botswana Harvard Health Partnership, Gaborone, Botswana; Department of Clinical Research, Faculty of Infectious and Tropical Diseases, London School of Hygiene and Tropical Medicine, London, UK

**Keywords:** central nervous system infection, computed tomography, HIV, meningitis, neuroimaging

## Abstract

**Background:**

The role of computed tomography (CT) before lumbar puncture (LP) is unclear, with limited evidence for a causal link between LP and cerebral herniation or for the ability of CT to identify individuals at risk of herniation. The risks of LP delay or deferral are potentially greater in high–HIV prevalence, resource-limited settings; we analyzed data from such a setting to determine the impact of CT on time to LP and treatment, as well as mortality.

**Methods:**

Adults with suspected central nervous system (CNS) infection were enrolled prospectively into the Botswana National Meningitis Survey between 2016 and 2019. Inpatient mortality and clinical data including time of treatment initiation and CT were captured from medical records. Associations between preceding CT and outcomes were assessed using logistic regression.

**Results:**

LPs were performed in 711 patients with suspected CNS infection; 27% had a CT before LP, and 73% were HIV positive. Time from admission to LP and time from admission to appropriate treatment were significantly longer in patients who had a CT before LP compared with those who did not (2.8 hours and 13.2 hours, respectively). There was some evidence for treatment delays being associated with increased mortality; however, there was no significant difference in mortality between those who had or did not have CT.

**Conclusions:**

Patients who had a CT had delays to diagnostic LP and initiation of appropriate treatment; although treatment delays were associated with increased mortality, our observational study could not demonstrate a causal association between delays in diagnosis and treatment introduced by CT and mortality.

Central nervous system (CNS) infections are a major cause of morbidity and mortality globally, with the highest disease burdens in resource-limited settings [1]. Rapid diagnosis and initiation of treatment are essential to improve CNS infection outcomes [2]. Lumbar puncture (LP) is the key investigation for guiding management. However, clinicians are often reluctant to perform LPs due to concerns about precipitating cerebral herniation in patients with increased intracranial pressure (ICP) [3–5].

Computed tomography (CT) is often requested before performing LPs to identify imaging appearances that may contraindicate LP. Evidence supporting the association between LP and cerebral herniation is limited, and while a temporal association has been demonstrated, no clear causal relationship has ever been established. There is no evidence that CT performed before LP improves outcomes in patients with suspected CNS infection [6, 7], and it is unclear whether CT accurately predicts those at risk of herniation or raised ICP; normal CT findings have been seen in up to 80% of bacterial meningitis cases with brainstem herniation [3, 8–10]. Furthermore, CT before LP has been shown to delay diagnostic LP, often resulting in administration of antimicrobials before LP and a subsequent reduction in yield from cerebrospinal fluid (CSF) culture [6, 11, 12].

Despite lack of evidence for benefit, in resource-rich countries most patients have CT before LP; 94% of UK patients had CT before LP, with only 17% having an indication based on national guidelines [13]. This results in large amounts of potentially unnecessary imaging, adding delays, and cost to management. In 2009, Sweden introduced guidelines limiting the number of indications for CT in patients with suspected CNS infection; a series of studies subsequently demonstrated that prompt LP without preceding CT resulted in earlier treatment initiation and more favorable outcomes in bacterial meningitis [7, 14].

Several guidelines recommend when to perform CT in patients with suspected CNS infection based on clinical features predicting the presence of radiological abnormalities [15–17]. However, most originate from countries with rapid access to CT and do not consider the distinct challenges in resource-limited settings where the burden of meningitis is greater and mortality higher [1]; the need for prompt diagnosis and appropriate antimicrobial therapy is therefore crucial [18, 19]. High HIV prevalence in many resource-limited regions widens the differential diagnosis for suspected CNS infection, making diagnostic LP essential for guiding treatment, but also raises the likelihood of pathology that may contraindicate LP. CT availability is extremely constrained in resource-limited settings, often only available in specialized hospitals, and even then, with limited access. These factors mean the risk–benefit balance of delaying LPs to perform CT is potentially very different than in resource-rich settings. As such, adoption of guidelines from resource-rich countries may not be appropriate or feasible and could result in unnecessary delay or deferral of LP, leading to poorer outcomes [20].

Data from resource-limited high–HIV prevalence settings describing the impact of CT on treatment delay and mortality in suspected CNS infection are limited. We analyzed data from a prospective cohort of adults presenting with suspected CNS infection to a national referral hospital in Botswana, where HIV prevalence in adults aged 15–49 is 18.6% [21], describing the use of CT and determining the impact of CT on time to LP, treatment initiation, and in-hospital mortality.

## METHODS

### Study Setting

The Botswana National Meningitis Survey is an ongoing meningitis surveillance study [22]. Clinical and laboratory data are collected prospectively from all patients with CSF submitted to the microbiology laboratory at the national public referral hospital, Princess Marina Hospital (PMH) in Gaborone. We analyzed data collected between November 2016 and September 2019. Botswana has a relatively well-resourced health care infrastructure compared with other low- and middle-income countries in Sub-Saharan Africa, with CT available and provided free of charge to citizens at PMH.

### Study Methodology

All adults aged 18 years or older with CSF submitted to the microbiology laboratory were included. Repeat LPs during the same or a subsequent admission and LPs performed for reasons other than suspected CNS infection were excluded. LPs were performed by treating clinicians, and CSF analysis was performed as previously described [23]. Patients were followed by the study team during admission. Laboratory data including timing of CSF collection were captured from laboratory records. Clinical data were recorded from the patients’ medical notes and drug administration charts. Final diagnoses were assigned by 2 members of the study team (J.M./C.W.) based on standardized case definitions (Supplementary Table 1), with arbitration by J.N.J. in cases of disagreement. Appropriate CNS infection treatment was defined as antibiotics (third-generation cephalosporins or other accepted bacterial meningitis treatment regimens) in confirmed or probable bacterial meningitis, antifungal treatment with amphotericin B–based therapy in confirmed cryptococcal meningitis, antituberculous therapy in possible, probable, or definite tuberculous meningitis (TBM) cases [24], and antiviral therapy in confirmed herpes simplex virus (HSV)–1, HSV-2, or varicella zoster virus CNS infection when there was clinical suspicion of encephalitis.

CT was performed upon the request of treating clinicians with no input from the study team. CT timing was determined from scan image time stamps, CT scanner electronic log, or patients’ medical notes. Published consensus guidelines for performing CT before LP have been developed for PMH (Supplementary Table 2) [25]. Reporting of CT scans was performed by consultant physicians or by radiologists upon clinician request. Potential contraindications to LP were defined as lateral shift of midline structures, hydrocephalus, any intracranial lesion regardless of size or mass effect, cerebral edema, or intracranial bleed.

Patients were followed up to hospital discharge. The study team had no role in the management of recruited patients. All data were anonymized, linked through unique study ID numbers, and uploaded onto a secure electronic data capture system [26, 27].

Clinical and CSF characteristics were compared for patients who received CT before LP and those who did not; continuous variables were compared using Wilcoxon rank-sum testing and categorical variables using the chi-square test for proportions. Time to appropriate CNS infection antimicrobial initiation was dichotomized at clinically meaningful time points depending on the pathogen. Multivariate regression analysis was performed to determine associations between the key exposure of interest (CT before LP) and outcome variables (time from admission to LP and to appropriate CNS infection–targeted antimicrobial initiation, inpatient mortality). Sensitivity, specificity, and positive and negative predictive values for the detection of potential radiological contraindication to LP on CT were calculated for 3 guidelines. To adjust for differences in the severity of presentations, a composite value based on a standardized scoring system that utilizes abnormalities in physiological parameters was included in adjusted analyses (Supplementary Table 3) [28]. Fundoscopy was infrequently performed, and therefore papilloedema was not included in adjusted Infectious Diseases Society of America (IDSA) guidelines for this analysis.

### Ethics

Institutional review board approval was in place from the Health Research Development Council (Botswana Ministry of Health and Wellness), London School of Hygiene and Tropical Medicine, University of Botswana, and PMH.

## RESULTS

**Figure 1.**
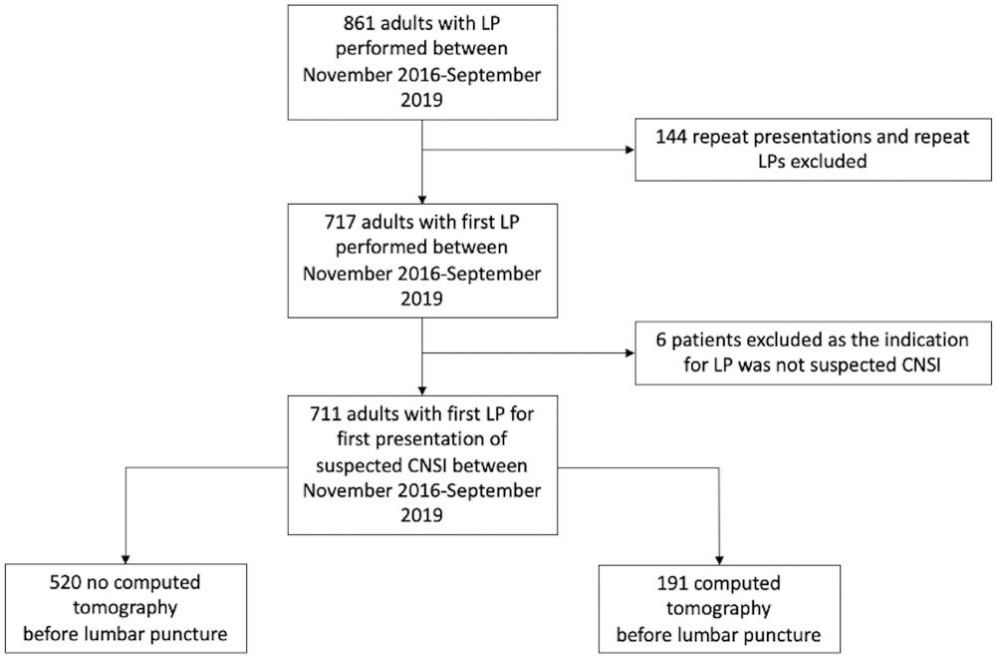
Screening, exclusion and analysis populations. Abbreviations: CNSI, central nervous system infection; LP, lumbar puncture.

Between November 2016 and September 2019, 861 LPs were performed on adults at PMH; 144 repeat LPs and 6 LPs performed for reasons other than suspected CNS infection were excluded. Data from the remaining 711 adults with suspected CNS infection were included for analysis (Figure 1).

### Study Population

The median age (interquartile range [IQR]) was 39 (32–48) years; 378/711 (53%) were male. HIV status was known in 669/711 (94.1%) patients, and 77% (519/669) of participants with known HIV status were HIV positive (Table 1).

**Table 1.** Clinical and Laboratory Characteristics of Patients With Suspected CNS Infection and Proportion of Patients who Received Appropriate Treatment During Admission

	All Patients (n = 711)	No Preceding CT Before LP (n = 520)	CT Performed Before LP (n = 191)	*P* Value
Median age (IQR), y	39 (32–48)	39 (32–47)	40 (32–54)	.16
Male gender, No. (%)	378 (53.2)	276 (53.1)	102 (53.4)	.94
HIV positive, No. (%)^a^	519 (77.6)	396 (81.7)	123 (66.9)	**<.001**
Median CD4 (IQR),^b^ cells/mm^3^	190 (55–412)	172 (49–390)	237 (80–480)	.05
GCS				
Normal GCS (GCS 15)	362 (50.9)	301 (57.9)	61 (31.9)	**<.001**
Minimally impaired GCS (GCS 13–14)	195 (27.4)	127 (24.4)	68 (35.6)	**.003**
Moderately impaired GCS (GCS 9–12)	103 (14.5)	62 (11.9)	41 (21.5)	**.001**
Severely impaired GCS (GCS ≤8)	7.2 (51)	30 (5.8)	21 (11.0)	**.02**
History of seizure, No. (%)	113 (15.9)	59 (11.4)	54 (28.3)	**<.001**
Focal neurology, No. (%)	95 (13.4)	45 (8.7)	50 (26.2)	**<.001**
Indication for imaging before LP based on local guidelines, No. (%)^c^ (GCS <15 or history of seizure or focal neurology)	421 (59.2)	262 (50.4)	159 (83.3)	**<.001**
CSF pleocytosis, No. (%)	201 (28.3)	156 (30.0)	45 (23.6)	.08
First review by clinician outside of routine working hours (1900–0900), No. (%)	321 (45.2)	217 (41.7)	104 (54.5)	.17

*P* values compare patients with no computed tomography performed before LP and patients with preceding computed tomography, calculated using Wilcoxon rank-sum for medians and the chi-square test for proportions. *P* values <.05 are displayed in bold.

Abbreviations: CNS, central nervous system; CSF, cerebrospinal fluid; CT, computed tomography; GCS, Glasgow Coma Scale; IQR, interquartile range; LP, lumbar puncture.

^a^HIV status known in 669 patients; 485 with no CT before LP and 184 with CT before LP. One hundred nine new diagnoses of HIV were made on presentation to Princess Marina Hospital, 21.2% (84/109) in patients with no CT before LP and 20.3% (25/109) in those with CT before LP.

^b^CD4 known in 385/519 HIV-positive patients.

^c^Based on consensus guidelines from Princess Marina Hospital, Gaborone [25].

CT before LP was performed in 191/711 (26.9%) patients. Those with CT before LP were more likely to have a reduced Glasgow Coma Scale score, recent seizures, and focal neurological deficits (Table 1). Age and sex were similar in those who did and did not have CT, but those who had CT had a lower HIV prevalence (66.9% [123/184] vs 81.7% [396/485]; *P* < .001). There was no significant difference in the proportion of patients with pleocytosis between those who did and did not have CT, and similar proportions received treatment for confirmed CNS infection or empiric antibiotics in both groups.

Based on local guidelines (Supplementary Table 2), an indication for CT before LP was present in 83.3% (159/191) of those who had CT before LP and 50.4% (262/520) of those who did not have CT; a smaller proportion of HIV-positive individuals had an indication for CT before LP than patients who were not HIV positive (58.4% [303/519] vs 71.3% [107/150]; *P* = .004).

### Impact of CT on Time to LP and Treatment

**Figure 2.**
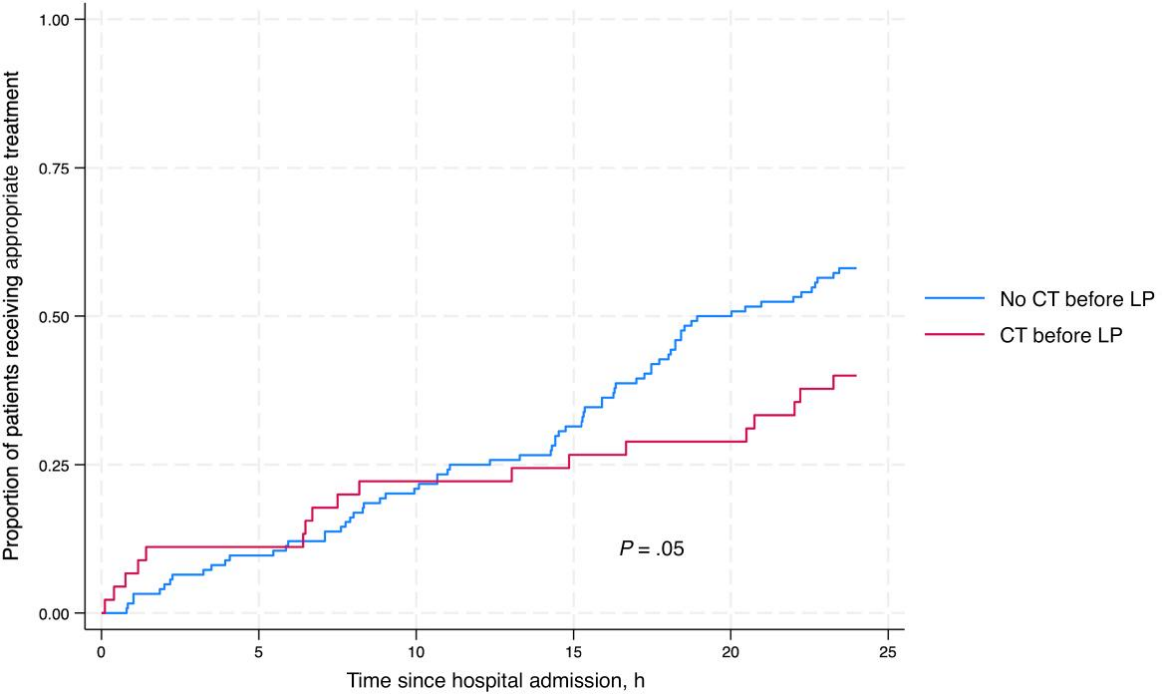
Kaplan-Meier analysis comparing receipt of appropriate antimicrobial treatment among patients with and without CT before LP in the first 24 hours after hospital admission in patients with microbiologically confirmed CNS infection or TB meningitis diagnosed through a uniform case definition. Abbreviations: CNS, central nervous system; CT, computed tomography; LP, lumbar puncture; TB, tuberculosis.

CT before LP was associated with a longer median (IQR) duration from admission to LP (11.8 [7.0–44.2] hours compared with 9.0 [4.6–25.0] hours; *P* = .004) (Table 2). Overall, there was a longer delay from admission to appropriate CNS infection treatment (IQR) in patients who had CT before LP (32.1 [16.7–96.3] hours vs 18.9 [11.1–44.1] hours; *P* = .05) (Figure 2), and a greater proportion of patients received appropriate CNS infection–targeted antimicrobial treatment within the first 24 hours if they did not undergo preceding CT (58.5% vs 40.0%; *P* = .04) (Table 2).

**Table 2.** Comparison of Time to Treatment Among Patients who Did and Did Not Undergo CT Before LP

	All Patients (n = 711)	No Preceding CT Before LP (n = 520)	CT Performed Before LP (n = 191)	*P* Value
Median time from admission to LP (IQR), h	9.8 (5.6–29.9) n = 484	9.0 (4.6–25.0) n = 327	11.8 (7.0–44.2) n = 157	**.004**
Median time to appropriate treatment (IQR), h	22.6 (13.1–49.6) n = 168	18.9 (11.1–44.1) n = 123	32.1 (16.7–96.3) n = 45	.05
Median time from admission to antibiotics in patients with confirmed bacterial meningitis or neutrophilic CSF pleocytosis (IQR), h	5.9 (1.4–8.3) n = 29	5.5 (1.9–8.9) n = 19	6.4 (1.2–8.2) n = 10	.87
Median time from admission to cryptococcal meningitis treatment (IQR), h	24.1 (16.3–38.5) n = 88	22.1 (15.4–36.0) n = 66	32.7 (22.2–68.0) n = 22	**.02**
Median time from admission to antituberculous treatment in patients with “possible,” “probable,” or “definite” tuberculous meningitis and no alternative CNS infection diagnosis (IQR), h	48.7 (18.4–129.2) n = 50	42.7 (18.0–112.3) n = 37	128.1 (37.0–198.0) n = 13	.09
Appropriate treatment started during admission, n/N (%)^a^	189/240 (78.8)	141/185 (76.2)	48/55 (87.3)	.60
Appropriate treatment administered in first 24 h of admission, % (n/N)^b^	53.6 (90/168)	58.5 (72/123)	40.0 (18/45)	**.04**

*P* values <.05 are displayed in bold.

Abbreviations: CNS, central nervous system; CT, computed tomography; HSV, herpes simplex virus; IQR, interquartile range; LP, lumbar puncture; VZV, varicella zoster virus.

^a^Appropriate treatment by organism: Cryptococcal meningitis treatment was given in 101/119 patients with cryptococcal meningitis, no treatment given in 9 patients, and treatment status unknown in 9 patients. Antibiotics were given in 31/31 in patients with confirmed or probable bacterial meningitis. Antiviral treatment was given in 3/3 patients with viral CNS infection due to HSV-1, HSV-2, or VZV. There was 1 VZV/cryptococcal meningitis coinfection that received both cryptococcal meningitis treatment and antiviral treatment. The first treatment given was cryptococcal meningitis treatment, and this was used for all analyses.

^b^Only 1 patient with viral meningitis had time to treatment data available. This was included in the time to appropriate treatment analysis but not presented separately.

TBM treatment was given in 54/71 patients with possible, probable, or definite TBM based on a uniform case definition [24] and no confirmed alternative CNS infection, no treatment was given in 17 patients, and treatment status was unknown in 1 patient.

Empiric CNS infection–targeted antibiotics were given in 81.1% of all cases regardless of final diagnosis, most commonly third-generation cephalosporins (76.8% of all antibiotic prescriptions) started at a median (IQR) of 8 (3.2–17.1) hours after admission (before LP in 58.1% of patients), and time to appropriate antimicrobial treatment (IQR) was not significantly longer in patients with confirmed or suspected bacterial meningitis who had preceding CT compared with those who did not (6.4 [1.2–8.2] hours vs 5.5 [1.9–8.9] hours; *P* = .87). Appropriate treatment was initiated later in patients who underwent CT with cryptococcal meningitis (32.7 [22.2–68.0] hours vs 22.1 [15.4–36.0] hours; *P* = .024) and TBM (128.1 [37.0–198.0] hours vs 42.7 [17.7–112.3] hours; *P* = .09).

### Impact of Treatment Delay on Mortality

Outcome was known for 687/711 (96.7%) patients; all-cause in-hospital mortality was 27% (184/687). Mortality in patients with a final diagnosis of CNS infection (Supplementary Table 1) and a known outcome was 34.4% (56/163); mortality was 27.5% (11/40) in those who received appropriate treatment within 12 hours of admission and 35.8% (45/123) in those receiving treatment >12 hours after admission; however, this did not reach statistical significance (adjusted OR, 1.66; 95% CI, 0.66–4.18) (Table 3). Limiting analysis to individuals with confirmed or probable bacterial meningitis, mortality in patients who received antibiotics within 12 hours after admission was 30.4% (7/23), and it was 40.0% (2/5) in those receiving treatment >12 hours after admission (adjusted OR, 2.22; 95% CI, 0.17–29.98); among those with cryptococcal meningitis, mortality was 18.2% (2/11) in those treated within 12 hours of admission and 32.4% (24/74) in those receiving treatment >12 hours after admission (adjusted OR, 1.41; 95% CI, 0.24–8.31), although these associations were not statistically significant. Delays >24 hours from admission in starting antituberculous treatment were significantly associated with increased mortality in patients with possible, probable, or definite TBM, 48.4% (15/31) vs 23.5% (4/17; adjusted OR, 6.89; 95% CI, 1.01–46.99).

**Table 3.** Table Describing Mortality in Patients who Received Appropriate Treatment for CNS Infection Within 12 Hours After Admission, Amphotericin B–Based Treatment for Cryptococcal Meningitis Within 12 Hours After Admission, and Appropriate Tuberculous Meningitis Treatment Within 24 Hours; Logistic Regressions Analysis Adjusting for Age, Sex, Glasgow Coma Score, and Composite NEWS

Time From Admission to Therapy	Mortality, % (No.)	Crude Odds Ratio for Mortality (95% CI, *P* Value)	Adjusted Odds Ratio for Mortality (95% CI, *P* Value)
Appropriate therapy for all CNS infection			
Under 12 h	27.5 (11/40)	-	-
After 12 h	35.8 (45/123)	1.52 (0.69–3.35, .29)	1.66 (0.66–4.18, .28)
Antibiotics in confirmed bacterial meningitis or probable bacterial meningitis with neutrophilic pleocytosis and no confirmed CNS infection			
Under 12 h	30.4 (7/23)	…	…
Over 12 h	40.0 (2/5)	1.52 (0.20–11.72, .68)	2.22 (0.17–29.98, .55)
Amphotericin B–based cryptococcal meningitis treatment			
Under 12 h	18.2 (2/11)	-	-
Over 12 h	32.4 (24/74)	2.16 (0.43–10.98, .34)	1.41 (0.24–8.31, .71)
Antituberculous treatment in patients with possible, probable, or definite tuberculous meningitis			
Under 24 h	23.5 (4/17)	-	-
Over 24 h	48.4 (15/31)	3.05 (0.77–12.10, .10)	6.89 (1.01–46.99, .05)

Abbreviation: CNS, central nervous system.

### Impact of Preceding CT on Inpatient Mortality

Limiting the analysis to those with a final diagnosis of CNS infection, including those with CSF pleocytosis suggestive of CNS infection but without definitive microbiology, inpatient mortality for patients without an indication for preceding CT was 20% (21/105). In those patients without an indication for CT, mortality was 18.2% (2/11) in patients who had CT compared with 20.2% (19/94) in those without (adjusted OR, 0.92; 95% CI, 0.17–4.81). For patients with an indication for CT before LP, mortality was 41.2%; it was 41.7% (25/73) in those who had CT compared with 41.0% (48/117) in those who did not (adjusted OR, 1.01; 95% CI, 0.50–2.08).

### Detection of Contraindications to Lumbar Puncture on CT

Potential radiological contraindications were identified on 24.5% (40/163) of CT scans performed before LP among those patients with an available report. Among patients who had an indication for imaging based on local guidelines, 25.7% (35/136) had a potential radiographic contraindication to LP compared with 18.5% (5/27) who did not have an indication for imaging (*P* = .43). There was a trend toward higher inpatient mortality in the group with potential radiological contraindications when compared with the group without, 39.5% and 26.0%, respectively; however, this did not reach statistical significance (adjusted OR, 2.02; 95% CI, 0.93–4.44) (Table 4).

**Table 4.** Associations Between Clinical and Laboratory Features Included in National or International Guidelines, CSF Analysis, Final Diagnosis and Mortality, and the Presence or Absence of Potential Radiological Contraindications to LP; Radiological CI Defined as Presence of Hydrocephalus, Intracranial Lesion, Cerebral Edema, Midline Shift or Intracranial Bleed; Patients Without a CT Report Were Not Included in the Analysis

	No Radiological CI to LP Identified on CT Head (n = 123)	Potential Radiological CI to LP Identified on CT Head (n = 40)	*P* Value
Final diagnosis of:			
Cryptococcal meningitis, No. (%)	16 (13.0)	5 (12.5)	.93
Possible/probable/definite tuberculous meningitis, No. (%)	9 (7.3)	9 (22.5)	.008
Viral meningitis, No. (%)	1 (0.8)	2 (5)	.09
Confirmed or probable bacterial meningitis, No. (%)	7 (5.7)	2 (5)	.87
Mortality	32/123 (26.0)	15/38 (39.5)	
	…	Crude OR for mortality, 1.86 (95% CI, 0.86–4.02)	.11
	…	Adjusted OR^a^ for mortality, 2.03 (95% CI, 0.93–4.44)	.08

Outcome data were missing for 2 patients.

Abbreviations: CI, confidence interval; CSF, cerebrospinal fluid; CT, computed tomography; LP, lumbar puncture; OR, odds ratio.

^a^Adjusted for age and sex.

Using data from all individuals undergoing CT, the sensitivity and specificity and positive and negative predictive values of different guidelines in identifying potential radiological contraindications are described in Supplementary Table 4.

## DISCUSSION

CT before LP in a high–HIV prevalence African setting led to significant delays to diagnostic LP and initiation of appropriate treatment. There was some evidence that delays in treatment were associated with increased mortality in patients with confirmed CNS infection; however, we were unable to determine a direct link between imaging and mortality, potentially due to the observational nature of the study, with imaging requested at the discretion of responsible clinicians, and significant confounding by indication. Clinical criteria to determine the need for CT before LP had poor sensitivity and specificity for subsequent detection of radiological criteria that would potentially contraindicate LP.

Our data are consistent with studies from high-income settings showing that performing CT in individuals with suspected CNS infection delays LP and definitive diagnosis. However, differences in CNS infection epidemiology in high-income settings, with a predominance of bacterial and self-limiting viral meningitis and a high frequency of empiric antibiotic administration, mean that delays in LP are less likely to delay effective treatment [10, 11, 14, 29], in contrast to settings where the spectrum of common CNS infections is broader, many requiring specific treatments including TB therapy and antifungals. In high–HIV prevalence settings in Sub-Saharan Africa, cryptococcal and TB meningitis account for 70%–90% of CNS infection, with bacterial meningitis accounting for only 8%–30% [22, 30, 31], highlighting why context-specific guidance regarding CT before LP is needed.

In our study, LPs were performed nearly 3 hours later in patients who had CT, with considerable knock-on delays in the subsequent initiation of targeted antimicrobial therapy. The impact of CT on treatment initiation time varied by condition, with a degree of delay seen for all major pathologies. Empiric antibiotics were administered to most patients, limiting the impact of imaging on time to appropriate antimicrobials with confirmed or probable bacterial meningitis; longer delays were seen with antituberculous, antiviral, and cryptococcal meningitis treatment, which are more complex and typically only started once diagnostic information from LPs is available. The delays to treatment initiation associated with CT in these cases were considerably longer than could be accounted for by simply the delays to LP. This may be due to hospital workflows where treatment decisions are made on daily ward rounds; if diagnostic information is unavailable at the initial postintake rounds, then treatment decisions are deferred until the next medical review, often the next day.

Previous studies have demonstrated that delay to the administration of appropriate treatment is associated with increased mortality [32–36]; antibiotic delays in bacterial meningitis significantly increase unfavorable outcomes, and TBM treatment delays beyond 3 days lead to a 70% mortality increase [35, 36]. Our data corroborated this, with delays of >24 hours to treatment initiation in TBM being associated with a significant increase in mortality on multivariate regression analysis. Increased mortality with delays to initiation of therapy was seen in other conditions, but our sample size was too small to demonstrate significant effects.

We were unable to determine whether there was a causal link between CT-induced delays in LP and treatment and increased mortality due to limitations in our study design. We only included patients who had an LP; therefore patients with suspected CNS infection who did not have an LP due to CT findings would not be captured, meaning we cannot comment on overall percentages of individuals with imaging abnormalities in the population presenting with a clinical syndrome compatible with a CNS infection. The selection of patients who received CT was not random and was chosen by the treating physicians based on clinical features; those who underwent CT are likely to have presented with more severe illness; although we tried to account for differences in disease severity based on recorded variables and adjust for these in our analyses, it is inevitable that several markers of severity will not have been captured and fully accounted for, leaving residual confounding by indication.

Guidelines on the use of CT in CNS infection management in resource-limited settings are not widely available. Although consensus guidelines have been published for our study site, adherence was poor, with over half of those without imaging having a guideline indication for CT. Furthermore, these guidelines were poorly predictive of radiologic contraindications. Most patients with an indication for CT who received CT had no radiographic contraindication to immediate LP, and 18.5% of patients with no indication for imaging who had a CT scan had a potential contraindication to LP. The sensitivity of IDSA guidelines for detecting abnormalities in this cohort would have been 95%, the highest among guidelines tested [37]. However, using immunosuppression as an indication for CT, as in the IDSA guidance, would be impractical to implement in Botswana, where 73% (519/711) of our study population was known to be HIV positive. If IDSA guidelines were followed appropriately, this would have meant an additional 446 patients would have had CT before LP in this cohort (Supplementary Table 4) in a center where radiology is already operating at maximum capacity. Increased CT scanning in patients with suspected CNS infection would also have significant resource implications alongside potentially unnecessary delays to LP and treatment.

People with HIV (PWH) were significantly less likely to have an indication for CT based on local guidelines (or to undergo CT), in contrast to most guidelines where immunosuppression is an indication for CT. The relatively low level of CT being performed among PWH might be explained by the high rates of cryptococcal meningitis in this group [38]. Clinicians in high–HIV prevalence settings recognize that prompt LPs are a crucial, time-sensitive therapeutic intervention in cryptococcal meningitis to reduce ICP and may be less likely to request imaging when this is clinically suspected.

Our study had several further limitations. The standard of reporting of CTs was variable and not always by radiologists; as such, contraindications might have been under-recognized. The radiological contraindications to LP used in this study were pragmatically chosen to be easily identifiable by clinicians. Due to the real-world nature of the data with imaging requested at clinician discretion, and often not in line with local guidelines, we cannot determine whether CT before LP could prevent adverse consequences of LP; notably, it was observed that mortality was no different between the groups in our cohort with an indication for CT who did and did not undergo CT before LP. The outcomes of individuals with suspected CNS infection who had contraindications identified on CT and did not then undergo LP are not known. Finally, this study was performed in a relatively well-resourced hospital that has been involved in several clinical trials for CNS infection; therefore, these findings may not be generalizable to settings with additional resource limitations.

The risk–benefit balance of CT before LP in suspected CNS infections remains contentious. Our data demonstrate that performing CT before LP in a low-resource, high–HIV prevalence setting significantly increased time to diagnostic LP and time to appropriate treatment, with no evidence that CT before LP was associated with improved outcomes in individuals for whom current guidelines would recommend imaging. These findings need to be taken into consideration when developing context-appropriate guidelines to support clinician decision-making.

## Supplementary Data

Supplementary materials are available at *Open Forum Infectious Diseases* online. Consisting of data provided by the authors to benefit the reader, the posted materials are not copyedited and are the sole responsibility of the authors, so questions or comments should be addressed to the corresponding author.

## Supplementary Material

ofae565_Supplementary_Data
