# Supplementary material for: Computed Tomography of the Head Before Lumbar Puncture in Adults With Suspected Meningitis in High–HIV Prevalence Settings
Source: Open Forum Infect Dis. 2024 Sep 26;11(10):ofae565. doi: 10.1093/ofid/ofae565 (PMC11493084; doi:10.1093/ofid/ofae565)
Supplement: ofae565_Supplementary_Data [file ofae565_supplementary_data.docx]

**Supplementary material**

| **Viral CN**S infection   - Patient with symptoms consistent with CNS infection OR clinically suspected CNS infection - AND positive CSF PCR for a HSV-1, HSV-2 or VZV |
| --- |
| **Confirmed bacterial meningitis**   - Patient with symptoms consistent with meningitis OR clinically suspected meningitis - AND detection of an appropriate pathogen in CSF by PCR, culture, or Gram stain, OR detection of an appropriate pathogen in blood by PCR, culture, or Gram stain, with CSF pleocytosis. |
| **Probable bacterial meningitis**   - Patient with symptoms consistent with meningitis OR clinically suspected meningitis - AND neutrophillic pleocytosis with no other cause of CNS infection identified |
| **Cryptococcal Meningitis**   - Patient with symptoms consistent with meningitis OR clinically suspected meningitis - AND identification of *Cryptococcus neoformans/gattii* in CSF by culture, CrAg, India Ink or PCR |
| **Definite Tuberculous meningitis**   - Patient with symptoms and signs of meningitis including one or more of the following: headache, irritability, vomiting, fever, neck stiffness, convulsions, focal neurological deficits, altered consciousness, or lethargy - AND acid-fast bacilli seen in the CSF, OR identification of Mycobacterium tuberculosis in the CSF by culture or nucleic acid amplification test. |
| **Probable Tuberculous meningitis**   - Patient with symptoms and signs of meningitis including one or more of the following: headache, irritability, vomiting, fever, neck stiffness, convulsions, focal neurological deficits, altered consciousness, or lethargy. - AND, using Marais’ criteria, a total diagnostic score of 10 or more points (when cerebral imaging is not available) or 12 or more points (when cerebral imaging is available). At least 2 points should either come from CSF or cerebral imaging criteria. - AND alternative diagnoses excluded. |
| **Possible Tuberculous meningitis**   - Patient with symptoms and signs of meningitis including one or more of the following: headache, irritability, vomiting, fever, neck stiffness, convulsions, focal neurological deficits, altered consciousness, or lethargy. - AND, using Marais’ criteria, a total diagnostic score of 6-9 points (when cerebral imaging is not available) or 6-11 points (when cerebral imaging is available). - AND alternative diagnoses excluded. |

**Supplementary table 1** Meningitis case definitions for final diagnosis of central nervous system infection

| GCS <8 and/or posturing |
| --- |
| New onset seizures |
| Unexplained altered mental status not clearly caused by alternative diagnosis (e.g. hypoxia, hypotension, hypoglycaemia) |
| Focal neurologic findings |

**Supplementary table 2** *Summary of consensus guidelines for preceding CT*

|  | 3 | 2 | 1 | 0 | 1 | 2 | 3 |
| --- | --- | --- | --- | --- | --- | --- | --- |
| **HR** | <40 |  | 41-50 | 51-90 | 91-110 | 111-130 | >131 |
| **Systolic BP** | <90 | 91-100 | 101-110 | 111-219 |  |  | >220 |
| **RR** | <8 |  | 9-11 | 12-20 |  | 21-25 | >25 |
| **Temp** |  |  |  |  | >37.5 |  |  |
| **O2 Sats** |  | <88 | 88-92 | >92 |  |  |  |

**Supplementary table 3** *Composite scoring system based on routinely collected physiological parameters. Missing values were scored as zero.*

|  | **Sensitivity** | **Specificity** | **Positive predictive value** | **Negative predictive value** | **Percentage of patients that would meet criteria to require scan if guidelines followed** |
| --- | --- | --- | --- | --- | --- |
| **Adjusted IDSA guidelines**  *(GCS<15, HIV-positive, history of seizure or focal neurology)* | 95.0%  (95% CI 83-99%) | 6.5%  (95% CI 3-12%) | 24.8%  (95% CI 18-33%) | 80%  (95% 44-98%) | 89.6% (637/711) |
| **Princess Marina Hospital guidelines**  *(GCS<15, history of seizure or focal neurology)* | 87.5%  (95% CI 73-96%) | 17.9%  (95% CI 12-26%) | 25.7%  (95% CI 19-34%) | 81.5%  (95% 62-94%) | 59.2% (421/711) |
| **Adjusted Swedish guidelines**  *(GCS<6 or focal neurology)* | 37.5%  (95% CI 23-54%) | 74.0%  (95% CI 65-82%) | 31.9%  (95% CI 19-47%) | 78.4%  (95% CI 70-86%) | 14.6% (104/711) |

**Supplementary table 4** *Sensitvity, specificity, and positive and negative predictive value for the detection of potential radiological contraindications to lumbar puncture estimates for IDSA, PMH and Swedish guidelines. Fundoscopy was infrequently performed and therefore papilloedema was not included in the adjusted IDSA guidelines for this analysis*^43^
